# The association between circulating saturated fatty acids and thyroid function: results from the NHANES 2011−2012

**DOI:** 10.3389/fendo.2024.1405758

**Published:** 2024-10-07

**Authors:** Wei Zhao, Xinnan Peng, Yazhuo Liu, Shen Li, Xinyu Li, Zhengnan Gao, Cheng Han, Zizhao Zhu

**Affiliations:** ^1^ Department of Endocrinology and Metabolism, Central Hospital of Dalian University of Technology; Dalian Municipal Central Hospital, Dalian, Liaoning, China; ^2^ Department of Clinical Nutrition and Metabolism, Affiliated Zhongshan Hospital of Dalian University, Dalian, Liaoning, China; ^3^ Department of General Surgery, The Sixth People’s Hospital of Shenyang, Shenyang, Liaoning, China

**Keywords:** saturated fatty acids, thyroid function, thyroid hormones, lipid metabolism, National Health and Nutrition Examination Survey

## Abstract

**Background:**

Excessive saturated fatty acids (SFAs) are known to be detrimental to human health. Although the majority of research and dietary guidelines have focused on the intake of SFAs, there has been limited attention to the relationship between circulating SFA levels and hormonal regulation, such as that of thyroid hormones.

**Methods:**

To explore potential associations, we conducted an investigation with 579 participants from the National Health and Nutrition Examination Survey (NHANES) 2011–2012. Subgroup analyses and multivariable linear regression models were used to estimate the relationships between eleven distinct SFA concentrations and various thyroid parameters.

**Results:**

For 579 adults, subgroup analysis of thyroid stimulating hormone (TSH) revealed significant differences in nine specific SFAs and the total SFA levels (all *p* < 0.05). Furthermore, multivariable linear regression analysis identified positive correlations between certain SFAs and various parameters, including TSH, total triiodothyronine (TT3), free triiodothyronine (FT3), thyroid peroxidase antibodies (TPOAb), thyroglobulin antibodies (TgAb), thyroglobulin (Tg), the ratio of FT3 to free thyroxine (FT4) (FT3/FT4), and the thyrotroph T4 resistance index (TT4RI). Conversely, negative correlations were observed between certain SFAs and total thyroxine (TT4), FT4, the ratio of FT3/TT3, and the thyroid feedback quantile-based index (TFQI) (all *p* < 0.05).

**Conclusion:**

These findings collectively suggest associations between SFAs and thyroid parameters, highlighting the need for future studies to elucidate the underlying mechanisms of these interactions.

## 1 Introduction

Saturated fatty acids (SFAs) are simple linear chains of singly-bonded carbon atoms, widely found in both animal fats and vegetable oils. SFAs play essential physiological roles, serving as important components of cell membranes and maintaining cell structure and function. Additionally, SFAs regulate biochemical reactions and provide energy to the human body. However, excessive SFAs are linked to adverse health effects. Research has confirmed the role of SFAs in lipid metabolism; they reduce the activity of liver low-density lipoprotein (LDL) receptors, decrease the clearance of LDL particles in the blood, and lead to increased LDL cholesterol levels (1). SFAs can also induce cellular inflammation through Toll-like receptor activation, potentially accelerating the atherosclerotic process (2). While most literature on SFAs has focused on dietary content, there is a growing interest in serum SFA composition, which relates to recent dietary intake and also reflects absorption, endogenous synthesis, and metabolic status (3). Studies indicate that higher circulating SFAs can elevate the risks of metabolic syndrome, diabetes, cardiovascular disease, and mortality (4–6). Therefore, understanding the metabolic factors affecting circulating SFA concentrations is significant.

Thyroid hormones (THs) are established regulators of internal environment homeostasis and energy metabolism. Abnormalities in TH levels are associated with deleterious health consequences, raising the risk of atherosclerosis, metabolic syndrome, and nonalcoholic fatty liver disease (7, 8). THs influence the liver and adipose tissue by participating in lipolysis and adipogenesis pathways, stimulating the release of stored fat and dietary fat sources to produce circulating free fatty acids (FFAs), which are the main source of hepatic lipids. THs also regulate fatty acid transporter expression through TH receptors (9). Furthermore, they promote *de novo* lipogenesis by modulating the transcription of key genes involved in adipogenesis. In hypothyroidism, reduced intrahepatic β-oxidation leads to an accumulation of FFA substrates (10). We hypothesize that alterations in thyroid function may influence SFA metabolism. However, comprehensive population-based studies on this topic are limited.

This study aims to explore the potential correlations between various circulating SFAs and thyroid parameters, using data from the National Health and Nutrition Examination Survey (NHANES) from 2011 to 2012.

## 2 Materials and methods

### 2.1 Study population

**Figure 1:**
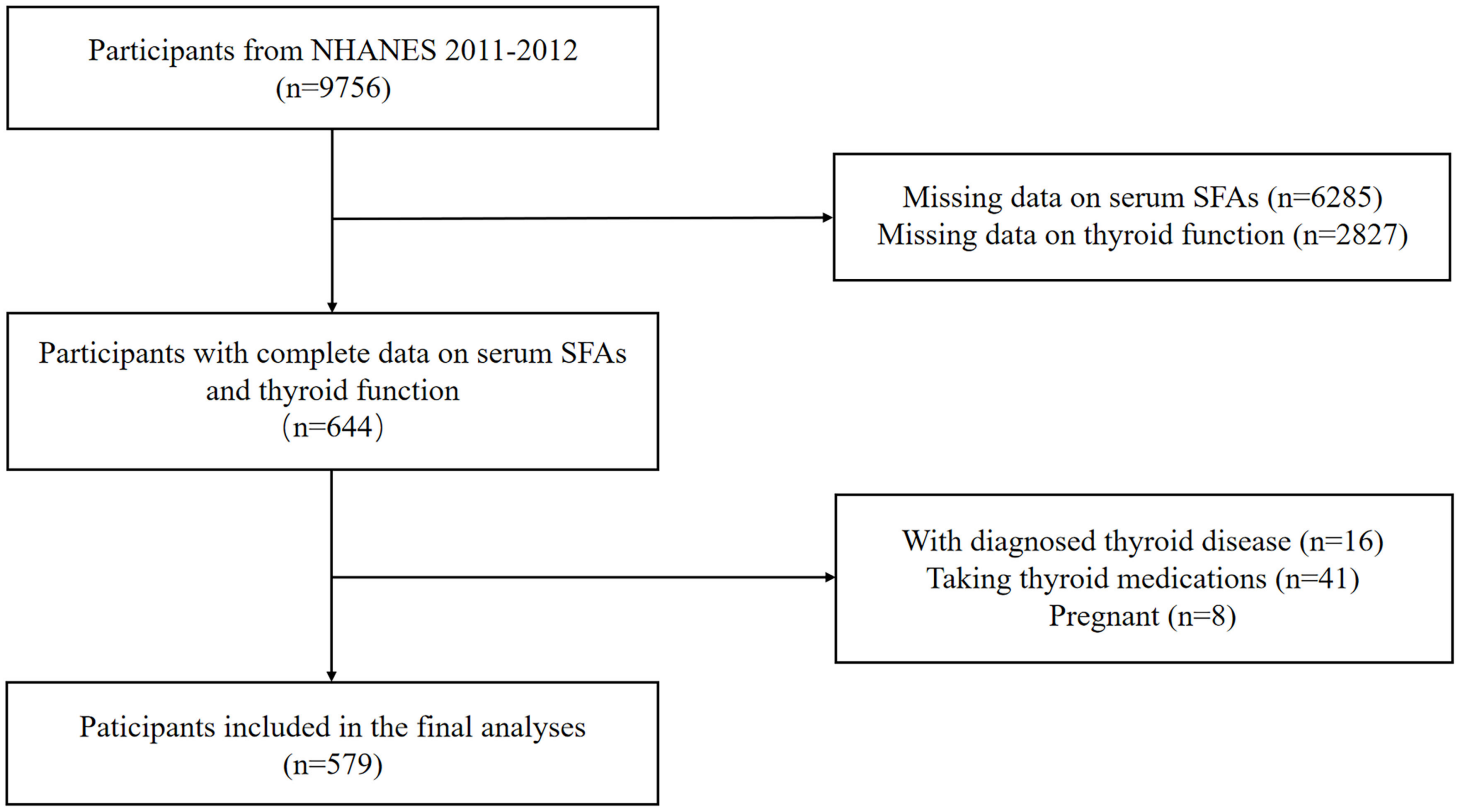
Flow chart of the participants selection process.

Data for this study was derived from the NHANES 2011-2012 dataset, a nationwide study approved by the Research Ethics Review Board of the National Center for Health Statistics (NCHS) to monitor the health and nutritional status of the American population. We included 644 participants who had complete data on serum or plasma SFAs and thyroid function measures. Individuals with thyroid diseases, those taking thyroid medications, or those who were pregnant were excluded. A sample size of 579 adults were included in the final analyses (**Figure 1**).

### 2.2 Thyroid function and SFAs measurement

Blood samples were collected from participants’ veins in the morning following an overnight fast, adhering to a standardized protocol. These serum samples were subsequently separated, stored, and frozen at −70°C. The dataset provided measurements of various thyroid function parameters, including thyroid stimulating hormone (TSH), total thyroxine (TT4), free thyroxine (FT4), total triiodothyronine (TT3), free triiodothyronine (FT3), thyroid peroxidase antibodies (TPOAb), thyroglobulin antibodies (TgAb), and thyroglobulin (Tg). Serum TSH levels were quantified using a third-generation two-site immunoenzymatic assay. A competitive binding immunoenzymatic assay was used to measure of T4 and T3. The assessment of TPOAb and TgAb levels involved a sequential two-step immunoenzymatic “sandwich” assay, while the measurement of Tg employed a simultaneous one-step “sandwich” method. Detailed information regarding the laboratory methods can be found in the **Supplementary Material** (**Supplementary Table S1**). Additionally, TSH levels were classified into subgroups as follows: decreased (< 0.4 μIU/mL), strict-normal (0.4-2.49 μIU/mL), low-normal (2.5-4.49 μIU/mL), and elevated (≥ 4.5 μIU/mL) (11). The ratios of FT3/FT4 and FT3/TT3 were calculated to assess peripheral TH metabolism (12, 13). Indices indicating central sensitivity to THs were computed using the following methods: thyroid feedback quantile-based index (TFQI) = cumulative distribution function (cdf) FT4 (pmol/L) + cdf TSH (μIU/mL) − 1; thyrotroph T4 resistance index (TT4RI) = FT4 (pmol/L) × TSH (μIU/mL); and TSH index (TSHI) = Ln TSH (μIU/mL) + 0.1345 × FT4 (pmol/L). Positive TFQI values, as well as elevated TT4RI and TSHI values, correspond to lower central sensitivity to THs (12).

The quantification of eleven SFAs was carried out using gas chromatography coupled with mass spectrometry. Quality control procedures were meticulously followed for all analyses, ensuring data quality and accuracy. Further details on laboratory methodology and quality assurance/quality control procedures can be found in the **Supplementary Material** (**Supplementary Table S1**). Moreover, the total SFAs value was obtained by summing the detected eleven SFAs.

### 2.3 Covariates

Potential confounders were selected based on clinical significance and previous studies (14, 15). Demographic factors included age, gender, ethnicity, education, and poverty index ratio (PIR) (≤ 1.30%, 1.31%-3.50%, or > 3.50%) (16). Lifestyle factors included smoking status, alcohol use, physical activity [insufficient, metabolic equivalent minutes per week (MET) ≤ 500; moderate, 500 < MET ≤1000; and vigorous, MET >1000] (17), and daily caloric intake (low: males < 2000 kcal, females < 1600 kcal; adequate: males 2000–3000 kcal, females 1600–2400 kcal; high: males >3000 kcal, females >2400 kcal) (18). Diabetes and hypertension were identified by meeting the clinical criteria, having self-reported physician diagnosis, or having a medical history of antidiabetic/antihypertensive (19). A history of atherosclerotic cardiovascular disease (ASCVD), including coronary heart disease, angina, heart attack or stroke (20), was also recorded. Body mass index (BMI) was classified as normal (< 25 kg/m^2^), overweight (25-29.9 kg/m^2^), and obesity (≥ 30 kg/m^2^) (18). Iodine nutritional status was defined based on urine iodine concentration (UIC) as insufficient (UIC < 100 µg/L), adequate (100-299 µg/L), and excessive (≥ 300 µg/L) (21). Biochemical analysis included alanine aminotransferase (ALT), aspartate aminotransferase (AST), alkaline phosphatase (ALP), and serum creatinine (SCr).

### 2.4 Statistical analysis

The normality of continuous variables was assessed using the Kolmogorov-Smirnov test. Continuous variables were presented as the mean ± standard deviation (SD) for normally distributed data, and as the median and interquartile range (IQR) for non-normally distributed data.

Categorical variables were presented as percentages. To examine differences in SFAs among TSH subgroups, the Kruskal-Wallis test was employed. Univariate and multivariate linear regression analyses were performed to evaluate the associations between predictor variables and SFAs. Covariates demonstrating significance at the *p* < 0.1 threshold in univariate analysis were subsequently included in the multivariate models. Non-normally distributed data of SFAs and thyroid function were log-transformed for modeling. Multiple imputations with chained equations were implemented to address missing data (22). The Variance Inflation Factor (VIF) was employed to assess collinearity among the variables. Statistical analyses were conducted using R version 4.3.1 (http://www.R-project.org). The level of significance was set at 0.05 (*p* < 0.05).

## 3 Results

### 3.1 Participant characteristics

**Table 1** presents characteristics of the 579 participants with average age 46.42 years. More than half of the adults (53.02%) were male, 33.85% were obese, over 40% were current or former smokers, and 74.95% were alcohol consumers. Among the participants, 19.86% had diabetes and 36.69% had hypertension, while approximately 10% were identified as having ASCVD. Nearly half of the subjects, according to UIC, exhibited moderate iodine nutrition.

**Table 1:** Characteristics of study participants.

Characteristics	Mean ± SD or Median (IQR)	No. of Participants
Demographic and lifestyle factors		
Age (Years)	46.42 ± 18.41	579
Gender (%)		
Male	53.02	307
Female	46.98	272
BMI category (Kg/m^2^) (%)		
BMI (Kg/m^2^)	28.49 ± 6.69	576
<25	34.38	198
25-29.9	31.77	183
≥30	33.85	195
Ethnicity (%)		
Non-Hispanic white	36.96	214
Non-Hispanic black	24.53	142
Mexican American	9.50	55
Other	29.02	168
Education (%)		
Less than high school	25.39	147
High school	48.19	279
More than high school	26.42	153
PIR category (%)		
≤1.30	37.50	198
1.31-3.50	35.23	186
>3.50	27.27	144
Smoking status (%)		
Current smoker	21.27	117
Former smoker	21.64	119
Non smoker	57.09	314
Alcohol use (%)		
Yes	74.95	380
No	25.05	127
Physical activity (%)		
Insufficient	14.41	66
Moderate	16.81	77
Vigorous	68.78	315
Daily calorie intake (%)		
Low	37.45	197
Adequate	42.97	226
High	19.58	103
Diabetes (%)		
Yes	19.86	115
No	80.14	464
Hypertension (%)		
Yes	36.96	214
No	63.04	365
ASCVD (%)		
Yes	9.44	52
No	90.56	499
Laboratory indicators		
ALT (U/L)	20.00 (16.00, 28.00)	578
AST (U/L)	23.00 (19.00, 27.00)	578
ALP (U/L)	64.00 (52.00, 78.00)	578
SCr (µmol/L)	74.30 (62.70, 84.80)	579
UIC category (µg/L) (%)		
<100	38.95	222
100-299	47.19	269
≥300	13.86	79

SD, standard deviation; IQR, interquartile range; BMI, body mass index; PIR, poverty index ratio; ASCVD, atherosclerotic cardiovascular disease; ALT, alanine aminotransferase; AST, aspartate aminotransferase; ALP, alkaline phosphatase; SCr, serum creatinine; UIC, urine iodine concentration.

### 3.2 Thyroid parameters and circulating SFAs levels

As demonstrated in **Table 2**, the median TSH, TT4, FT4, TT3, and FT3 concentrations of all subjects were 1.54 (IQR: 1.12, 2.26) μIU/mL, 7.93 (IQR: 6.97, 8.92) μg/dL, 10.70 (IQR: 9.80, 11.70) pmol/L, 117.00 (IQR: 104.00, 130.00) ng/dL, and 3.23 (IQR: 2.97, 3.46) pg/mL, respectively. The median FT3/FT4 and FT3/TT3 ratios were 0.30 (IQR: 0.27, 0.33) and 0.03 (IQR: 0.03, 0.03), respectively. The TFQI, TT4RI, and TSHI values were 0.015 ± 0.39, 16.48 (IQR: 12.18, 24.42), and 1.90 (IQR: 1.58, 2.29), respectively. Palmitic acid had the highest concentration [2660.00 (IQR: 2180.00, 3195.00) µmol/L] among the detected SFAs. The total median concentration of the eleven SFAs was 3649.66 (IQR: 3027.83, 4364.61) µmol/L.

**Table 2:** Thyroid function measures and SFA concentrations.

Serum Analyte	Mean ± SD or Median (IQR)
Thyroid function measures	
TSH (μIU/mL)	1.54 (1.12, 2.26)
TT4 (μg/dL)	7.93 (6.97, 8.92)
FT4 (pmol/L)	10.70 (9.80, 11.70)
TT3 (ng/dL)	117.00 (104.00, 130.00)
FT3 (pg/mL)	3.23 (2.97, 3.46)
TPOAb (IU/mL)	0.50 (0.18, 1.25)
TgAb (IU/mL)	0.60 (0.60, 0.60)
Tg (ng/mL)	10.75 (6.31, 17.38)
FT3/FT4	0.30 (0.27, 0.33)
FT3/TT3	0.03 (0.03, 0.03)
TFQI	0.015 ± 0.39
TT4RI	16.48 (12.18, 24.42)
TSHI	1.90 (1.58, 2.29)
SFA concentrations (µmol/L)	
Capric acid (C10:0)	1.12 (1.12, 2.31)
Lauric acid (C12:0)	6.87 (4.32, 13.00)
Myristic acid (14:0)	101.00 (69.20, 149.00)
Pentadecanoic acid (C15:0)	21.00 (15.40, 27.20)
Palmitic acid (16:0)	2660.00 (2180.00, 3195.00)
Margaric acid (C17:0)	29.30 (24.85, 35.20)
Stearic acid (18:0)	661.00 (561.0, 774.50)
Arachidic acid (20:0)	23.20 (19.80, 26.80)
Docosanoic acid (22:0)	54.55 (54.10, 75.50)
Tricosanoic acid (C23:0)	27.50 (23.10, 32.55)
Lignoceric acid (24:0)	56.10 (47.45, 65.20)
Sum SFAs	3649.66 (3027.83, 4364.61)

SD, standard deviation; IQR, interquartile range; TSH, thyroid stimulating hormone; TT4, total thyroxine; FT4, free thyroxine; TT3, total triiodothyronine; FT3, free triiodothyronine; TPOAb, thyroid peroxidase antibodies; TgAb, thyroglobulin antibodies; Tg, thyroglobulin; TFQI, thyroid feedback quantile-based index; TT4RI, thyrotroph thyroxine resistance index; TSHI, thyroid stimulating hormone index; SFA, saturated fatty acid.

### 3.3 SFAs levels based on TSH subgroups

As shown in **Table 3**, statistically significant differences were observed among the TSH subgroups in nine specific SFAs, including capric acid, lauric acid, myristic acid, pentadecanoic acid, palmitic acid, stearic acid, docosanoic acid, tricosanoic acid, lignoceric acid, and the sum of SFAs (all *p* < 0.05).

**Table 3:** SFA levels based on TSH subgroups.

SFAs	TSH (μIU/mL)				*p* value
	< 0.4	0.4-2.49	2.5-4.49	≥ 4.5	
Capric acid (C10:0)	1.12 (1.12, 1.12)	1.63 (1.12, 2.68)	1.12 (1.12, 2.20)	2.12 (1.12, 3.13)	< 0.001
Lauric acid (C12:0)	6.79 (2.58, 6.79)	7.84 (4.79, 14.20)	8.58 (4.63, 12.90)	16.30 (6.93, 33.60)	0.020
Myristic acid (14:0)	90.00 (70.20, 90.00)	113.00 (74.00, 175.00)	111.00 (79.10, 175.00)	152.00 (101.00, 246.00)	< 0.001
Pentadecanoic acid (C15:0)	18.60 (12.10, 18.60)	22.60 (16.90, 29.50)	22.90 (17.60, 32.70)	23.30 (17.30, 37.90)	< 0.001
Palmitic acid (16:0)	2460.00 (2460.00, 2910.00)	2750.00 (2260.00, 3410.00)	2850.00 (2420.00, 3310.00)	2930.00 (2690.00, 3540.00)	0.030
Margaric acid (C17:0)	27.60 (21.40, 31.50)	29.30 (25.10, 35.00)	31.30 (25.30, 39.30)	33.60 (27.50, 38.00)	0.080
Stearic acid (18:0)	592.00 (592.00, 794.00)	679.00 (567.00, 797.00)	652.00 (623.00, 884.00)	712.00 (698.00, 829.00)	0.010
Arachidic acid (20:0)	20.00 (20.00, 25.10)	23.50 (19.90, 27.00)	23.80 (19.60, 27.90)	22.90 (19.80, 27.00)	0.490
Docosanoic acid (22:0)	57.00 (51.00, 60.50)	67.40 (57.10, 78.50)	68.50 (58.10, 72.80)	62.10 (55.10, 67.80)	0.003
Tricosanoic acid (C23:0)	23.10 (20.60, 23.70)	28.30 (23.40, 33.60)	29.30 (24.60, 33.60)	25.40 (20.80, 28.90)	< 0.001
Lignoceric acid (24:0)	49.50 (49.50, 54.80)	57.60 (48.60, 69.30)	58.40 (49.30, 65.60)	52.40 (43.60, 55.20)	0.004
Sum SFAs	3359.01 (3359.01, 3953.10)	3799.15 (3156.56, 4611.50)	3932.82 (3314.01, 4648.21)	4095.83 (3684.97, 4946.92)	0.020

SFAs, saturated fatty acids; TSH, thyroid stimulating hormone.

### 3.4 Associations between SFAs and thyroid function measures

**Supplementary Table S2** displays univariate associations with SFAs, identifying age, gender, BMI, ethnicity, education, PIR, smoking, alcohol, calorie intake, diabetes, hypertension, ASCVD, and biochemical markers (ALT, AST, ALP, SCr, UIC) as significant at *p* < 0.1, which were included in multivariate models. VIFs ranging from 1.12 to 2.84 indicated no presence of multicollinearity.

As presented in **Table 4**; **Supplementary Table S3**, multivariable linear regression analysis revealed that myristic acid, pentadecanoic acid, margaric acid, and tricosanoic acid were positively associated with TSH as a continuous variable [β=0.103, 95% confidence interval (CI): 0.005 to 0.200; β=0.111, 95% CI: 0.046 to 0.176; β=0.071, 95% CI: 0.021 to 0.121; β=0.031, 95% CI: 0.004 to 0.059, respectively, all *p* < 0.05]. Furthermore, concentrations of four SFAs (lauric acid, myristic acid, pentadecanoic acid, and margaric acid) exhibited increasing trends from the lowest to highest TSH subgroups (all *p* for trend < 0.05). A negative relationship was revealed between capric acid and TT4 (β=-0.567, 95% CI: -1.097 to -0.038, *p* < 0.05). Capric, lauric, myristic, pentadecanoic, palmitic, stearic, tricosanoic, lignoceric acids, and the sum of SFAs were negatively associated with FT4 (β=-1.040, 95% CI: -1.686 to -0.395; β=-1.028, 95% CI: -1.626 to -0.430; β=-1.002, 95% CI: -1.374 to -0.629; β=-0.528, 95% CI: -0.800 to -0.256; β=-0.357, 95% CI: -0.566 to -0.148; β=-0.221, 95% CI: -0.420 to -0.023; β=-0.178, 95% CI: -0.324 to -0.032; β=-0.258, 95% CI: -0.425 to -0.092; β=-0.346, 95% CI: -0.543 to -0.148; respectively, all *p* < 0.05). Lauric, myristic, pentadecanoic, palmitic, margaric, stearic, arachidic acids, and the sum of SFAs were positively correlated with TT3 (β=0.756, 95% CI: 0.198 to 1.315; β=0.696, 95% CI: 0.336 to 1.056; β=0.569, 95% CI: 0.266 to 0.872; β=0.360, 95% CI: 0.072 to 0.648; β=0.396, 95% CI: 0.127 to 0.665; β=0.303, 95% CI: 0.051 to 0.556; β=0.164, 95% CI: 0.008 to 0.321; β=0.351, 95% CI: 0.083 to 0.620, respectively, all *p* < 0.05), while only lauric acid and myristic acid were significantly associated with increased FT3 (β=1.455, 95% CI: 0.314 to 2.596, *p* < 0.05; β=0.838, 95% CI: 0.138 to 1.539, *p* < 0.05). Additionally, there were positive associations between capric acid, lauric acid, myristic acid, arachidic acid, tricosanoic acid, lignoceric acid, and TPOAb (β=0.055, 95% CI: 0.004 to 0.105; β=0.050, 95% CI: 0.003 to 0.098; β=0.031, 95% CI: 0.002 to 0.060; β=0.014, 95% CI: 0.001 to 0.027; β=0.017, 95% CI: 0.008 to 0.025; β=0.009, 95% CI: 0.001 to 0.018, respectively, all *p* < 0.05), as well as tricosanoic acid and TgAb (β=0.020, 95% CI: 0.005 to 0.036, *p* < 0.05). Furthermore, margaric acid was significantly correlated with raised Tg (β=0.049, 95% CI: 0.001 to 0.098, *p* < 0.05).

**Table 4:** Multivariate regression analyses of SFAs in relation to TSH, TT4, FT4, TT3, and FT3.

SFAs	TSH (μIU/mL)	TSH subgroups (μIU/mL)					TT4 (μg/dL)	FT4 (pmol/L)	TT3 (ng/dL)	FT3 (pg/mL)
		< 0.4	0.4-2.49	2.5-4.49	≥ 4.5	*p* for trend				
	β (95% CI)									
Capric acid (C10:0)	0.071 (-0.099, 0.242)	Ref	0.690 (0.365, 1.014) ^*^	0.594 (0.280, 0.908) ^*^	0.848 (0.406, 1.290) ^*^	0.487	-0.567 (-1.097, -0.038) ^*^	-1.040 (-1.686, -0.395) ^*^	0.197 (-0.387, 0.780)	0.124 (-1.252, 1.500)
Lauric acid (C12:0)	0.128 (-0.053, 0.310)	Ref	0.545(-0.092, 1.183)	0.559 (0.002, 1.117) ^*^	1.134 (0.385, 1.883) ^*^	0.018	-0.331 (-0.871, 0.208)	-1.028 (-1.626, -0.430) ^*^	0.756 (0.198, 1.315) ^*^	1.455 (0.314, 2.596) ^*^
Myristic acid (C14:0)	0.103 (0.005, 0.200) ^*^	Ref	0.435 (0.227, 0.642) ^*^	0.475 (0.315, 0.635) ^*^	0.756 (0.475, 1.037) ^*^	0.021	-0.250 (-0.610, 0.109)	-1.002 (-1.374, -0.629) ^*^	0.696 (0.336, 1.056) ^*^	0.838 (0.138, 1.539) ^*^
Pentadecanoic acid (C15:0)	0.111 (0.046, 0.176) ^*^	Ref	0.437 (0.265, 0.609) ^*^	0.503 (0.305, 0.700) ^*^	0.562 (0.305, 0.819) ^*^	0.017	-0.041 (-0.285, 0.204)	-0.528 (-0.800, -0.256) ^*^	0.569 (0.266, 0.872) ^*^	0.532 (-0.070, 1.134)
Palmitic acid (C16:0)	0.049 (-0.008, 0.106)	Ref	0.180 (0.054, 0.306) ^*^	0.207 (0.030, 0.383) ^*^	0.353 (0.136, 0.570) ^*^	0.072	-0.120 (-0.306, 0.066)	-0.357 (-0.566, -0.148) ^*^	0.360 (0.072, 0.648) ^*^	0.413 (-0.063, 0.889)
Margaric acid (C17:0)	0.071 (0.021, 0.121) ^*^	Ref	0.187 (0.083, 0.291) ^*^	0.225 (0.137, 0.314) ^*^	0.360 (0.158, 0.562) ^*^	0.024	0.010 (-0.121, 0.141)	-0.109 (-0.303, 0.085)	0.396 (0.127, 0.665) ^*^	0.414 (-0.132, 0.960)
Stearic acid (C18:0)	0.040 (-0.019, 0.099)	Ref	0.094 (-0.013, 0.200)	0.105 (-0.058, 0.269)	0.262 (0.084, 0.440) ^*^	0.122	-0.095 (-0.239, 0.050)	-0.221 (-0.420, -0.023) ^*^	0.303 (0.051, 0.556) ^*^	0.316 (-0.115, 0.746)
Arachidic acid (C20:0)	0.027 (-0.015, 0.069)	Ref	0.172 (0.064, 0.279) ^*^	0.149 (-0.010, 0.308)	0.185 (0.050, 0.319) ^*^	0.824	-0.021 (-0.146, 0.104)	-0.090 (-0.264, 0.085)	0.164 (0.008, 0.321) ^*^	0.237 (-0.037, 0.583)
Docosanoic acid (C22:0)	0.008 (-0.031, 0.047)	Ref	0.208 (0.098, 0.319) ^*^	0.183 (0.079, 0.286) ^*^	0.126 (-0.019, 0.270)	0.459	-0.055 (-0.206, 0.096)	-0.171 (-0.362, 0.019)	0.062 (-0.079, 0.204)	-0.005 (-0.196, 0.186)
Tricosanoic acid (C23:0)	0.031 (0.004, 0.059) ^*^	Ref	0.298 (0.090, 0.505) ^*^	0.324 (0.165, 0.483) *	0.251 (0.029, 0.474) ^*^	0.334	-0.031 (-0.164, 0.103)	-0.178 (-0.324, -0.032) ^*^	0.073 (-0.100, 0.246)	0.008 (-0.195, 0.212)
Lignoceric acid (C24:0)	-0.007 (-0.044, 0.031)	Ref	0.147 (0.040, 0.254) ^*^	0.149 (0.058, 0.239) ^*^	0.051 (-0.084, 0.186)	0.548	-0.138 (-0.291, 0.015)	-0.258 (-0.425, -0.092) ^*^	0.019 (-0.117, 0.155)	-0.031 (-0.208, 0.145)
Sum SFAs	0.049 (-0.006, 0.103)	Ref	0.179 (0.066, 0.291) ^*^	0.201 (0.041, 0.361) ^*^	0.346 (0.149, 0.542) ^*^	0.070	-0.117 (-0.291, 0.057)	-0.346 (-0.543, -0.148) ^*^	0.351 (0.083, 0.620) ^*^	0.398 (-0.058, 0.855)

SFAs, saturated fatty acids; TSH, thyroid stimulating hormone; TT4, total thyroxine; FT4, free thyroxine; TT3, total triiodothyronine; FT3, free triiodothyronine; CI, confidence interval; *p < 0.05. Age, gender, BMI category, ethnicity, education, PIR, smoking status, alcohol use, daily calorie intake, diabetes, hypertension, ASCVD, ALT, AST, ALP, SCr, and UIC were adjusted. SFAs and thyroid function measures were log-transformed due to non-normally distributed.

### 3.5 Associations between SFAs and thyroid function indices

As shown in **Table 5**, multivariable linear regression analysis indicated that all eleven SFAs and total SFAs were positively associated with the ratio FT3/FT4, respectively (β=1.008, 95% CI: 0.515 to 1.500; β=1.542, 95% CI: 0.912 to 2.171; β=1.265, 95% CI: 0.905 to 1.625; β=0.704, 95% CI: 0.355 to 1.052; β=0.497, 95% CI: 0.280 to 0.715; β=0.270, 95% CI: 0.009 to 0.531; β=0.333, 95% CI: 0.108 to 0.558; β=0.194, 95% CI: 0.024 to 0.364; β=0.155, 95% CI: 0.003 to 0.308; β=0.167, 95% CI: 0.015 to 0.320; β=0.225, 95% CI: 0.085 to 0.364; β=0.481, 95% CI: 0.267 to 0.696, respectively, all *p* < 0.05). Myristic, pentadecanoic, palmitic, margaric and stearic acids, and total SFAs were negatively associated with FT3/TT3 (β=-0.713, 95% CI: -1.217 to -0.209; β=-0.665, 95% CI: -1.111 to -0.218; β=-0.380, 95% CI: -0.731 to -0.028; β=-0.439, 95% CI: -0.764 to -0.115; β=-0.337, 95% CI: -0.650 to -0.025; β=-0.373, 95% CI: -0.700 to -0.046, respectively, all *p* < 0.05). Furthermore, the levels of myristic acid and lignoceric acid were negatively associated with TFQI (β=-0.200, 95% CI: -0.379 to -0.020, *p* < 0.05; β=-0.081, 95% CI: -0.117 to -0.045, *p* < 0.05). Pentadecanoic acid and margaric acid exhibited positive associations with TT4RI (β=0.072, 95% CI: 0.001 to 0.143, *p* < 0.05; β=0.063, 95% CI: 0.014 to 0.112, *p* < 0.05). Additionally, no statistically significant correlations were identified between the SFA concentrations and TSHI.

**Table 5:** Multivariate regression analyses of SFAs in relation to thyroid function indices.

SFAs	FT3/FT4	FT3/TT3	TFQI	TT4RI	TSHI
	β (95% CI)				
Capric acid (C10:0)	1.008 (0.515, 1.500) ^*^	-0.261 (-0.726, 0.204)	-0.216 (-0.527, 0.096)	-0.005 (-0.194, 0.184)	-0.077 (-0.395, 0.242)
Lauric acid (C12:0)	1.542 (0.912, 2.171) ^*^	-0.487 (-1.120, 0.145)	-0.201(-0.481, 0.080)	0.052 (-0.132, 0.237)	0.017 (-0.260, 0.294)
Myristic acid (C14:0)	1.265 (0.905, 1.625) ^*^	-0.713 (-1.217, -0.209) ^*^	-0.200 (-0.379, -0.020) ^*^	0.029 (-0.078, 0.136)	-0.040 (-0.185, 0.104)
Pentadecanoic acid (C15:0)	0.704 (0.355, 1.052) ^*^	-0.665 (-1.111, -0.218) ^*^	-0.030 (-0.138, 0.078)	0.072 (0.001, 0.143) ^*^	0.056 (-0.042, 0.154)
Palmitic acid (C16:0)	0.497 (0.280, 0.715) ^*^	-0.380 (-0.731, -0.028) ^*^	-0.059 (-0.149, 0.031)	0.023 (-0.036, 0.082)	-0.019 (-0.083, 0.046)
Margaric acid (C17:0)	0.270 (0.009, 0.531) ^*^	-0.439 (-0.764, -0.115) ^*^	0.047 (-0.026, 0.120)	0.063 (0.014, 0.112) ^*^	0.063 (0.000, 0.125)
Stearic acid (C18:0)	0.333 (0.108, 0.558) ^*^	-0.337 (-0.650, -0.025) ^*^	-0.021 (-0.107, 0.066)	0.024 (-0.034, 0.082)	-0.007 (-0.075, 0.062)
Arachidic acid (C20:0)	0.194 (0.024, 0.364) ^*^	-0.129 (-0.355, 0.098)	0.009 (-0.061, 0.079)	0.020 (-0.023, 0.063)	0.004 (-0.053, 0.061)
Docosanoic acid (C22:0)	0.155 (0.003, 0.308) ^*^	-0.106 (-0.331, 0.119)	-0.034 (-0.085, 0.017)	-0.004 (-0.041, 0.032)	-0.006 (-0.054, 0.043)
Tricosanoic acid (C23:0)	0.167 (0.015, 0.320) ^*^	-0.117 (-0.412, 0.177)	-0.017 (-0.058, 0.024)	0.018 (-0.007, 0.043)	0.014 (-0.018, 0.047)
Lignoceric acid (C24:0)	0.225 (0.085, 0.364) ^*^	-0.048 (-0.292, 0.195)	-0.081 (-0.117, -0.045) ^*^	-0.026 (-0.059, 0.008)	-0.045 (-0.099, 0.010)
Sum SFAs	0.481 (0.267, 0.696) ^*^	-0.373 (-0.700, -0.046) ^*^	-0.054 (-0.142, 0.033)	0.023 (-0.034, 0.080)	-0.015 (-0.078, 0.047)

SFAs, saturated fatty acids; FT3, free triiodothyronine; FT4, free thyroxine; TT3, total triiodothyronine; TFQI, thyroid feedback quantile-based index; TT4RI, thyrotroph thyroxine resistance index; TSHI, thyroid stimulating hormone index; CI, confidence interval; *p < 0.05. Age, gender, BMI category, ethnicity, education, PIR, smoking status, alcohol use, daily calorie intake, diabetes, hypertension, ASCVD, ALT, AST, ALP, SCr, and UIC were adjusted. SFAs, FT3/FT4, FT3/TT3, TT4RI, and TSHI were log-transformed due to non-normally distributed.

## 4 Discussion

This investigation aimed to explore the relationship between various circulating SFAs and thyroid function in a sample of American adults. The results confirmed that palmitic acid and stearic acid were the most abundant SFAs in systemic circulation, consistent with these components being among the most commonly consumed SFAs in the human diet. Furthermore, in the process of endogenous synthesis, palmitic acid serves as the initial fatty acid product, which subsequently gives rise to stearic acid and other types of fatty acids through carbon chain extension and desaturation (3).

Moreover, our study revealed significant differences in the levels of nine specific SFAs among various TSH subgroups, predominantly showing an increase in SFA levels with elevated TSH. In the multivariate linear regression analyses, SFA levels overall demonstrated positive correlations with TSH, TT3, and the FT3/FT4 ratio. Conversely, they exhibited negative correlations with FT4 and the FT3/TT3 ratio.

THs play a crucial role in regulating life activities and energy metabolism. The hypothalamic-pituitary-thyroid (HPT) axis orchestrates the production of TSH, T3, and T4, maintaining homeostasis through a negative feedback mechanism (12). The intricate interaction between THs and lipid metabolism has been a subject of research for many years. TSH has been recognized as an activator of lipolysis. In addition to thyroid cells, TSH receptors are found in adipose tissue cells, hepatocytes, and ovaries, among others. The administration of recombinant human TSH to patients undergoing thyroidectomy and radioactive iodine therapy led to an increase in circulating levels of FFAs, indicating a distinct effect of TSH (23). Gagnon et al. (24) demonstrated that TSH-stimulated lipolysis in cultured human adipocytes was dependent on the protein kinase A (PKA) pathway, and this mechanism also increased FFA levels *in vivo*. These findings were in line with our observation that certain SFAs were correlated with increased TSH levels independently.

In addition, in our study, the majority of SFA concentrations were positively correlated with TT3 or FT3. It is acknowledged that the liver is a key target organ for THs, involved in the synthesis and metabolism of fatty acids. The *de novo* synthesis of FFAs involves a series of enzyme-catalyzed reactions, with key enzymes being acetyl-CoA carboxylase (ACC) and fatty acid synthase (FAS). ACC catalyzes the conversion of acetyl-CoA into malonyl-CoA. Under the catalytic action of FAS, the carbon chain of malonyl-CoA is elongated to form palmitic acid, which can be further converted into stearic acid and other fatty acids. T3, the biologically active form of TH, directly influences ACC and FAS. T3 enhanced the expression of ACC in rats via the TH receptor binding to the promoter of the ACC gene. In isolated chicken embryo hepatocytes, treatment with T3 increased the FAS mRNA. T3 was found to stimulate the expression of ACC and FAS via the transcription factor sterol regulatory element-binding protein 1 as well (25). It also enhanced expression of genes in glucose catabolism, supplying malonyl-CoA for *de novo* fatty acid synthesis (26). Additionally, T3 is involved in stimulating adaptive thermogenesis in adipose tissues. In the classic process of lipolysis, FFAs are produced from endogenous triglycerides by lipases such as patatin-like phospholipase domain containing 2 (PNPLA2), which is activated by PKA. In this regard, T3 was found to stimulate the expression of PNPLA2 mRNA in brown adipose tissue and the phosphorylation mediated by PKA, thereby enhancing lipase activity and strengthening its direct effect (27). These mechanisms offer a multifaceted interpretation of our findings that various SFAs, such as palmitic acid, stearic acid, and total SFAs, et cetera, were correlated with elevated T3 levels. However, it cannot be overlooked that the regulation of SFAs by THs is influenced by factors such as temperature and starvation, and is also subject to the synergistic effects of other hormones like insulin, glucagon, and cortisol (28). Therefore, the comprehensive effects under different environments and conditions require further in-depth study.

An intriguing aspect of our findings was the inconsistency in the correlation between SFA concentrations and T3 and T4, which warrants further investigation. Comprehensive indices, rather than single thyroid measures, can provide a more systematic assessment of the HPT axis status. For instance, FT4 is converted to FT3 by iodothyronine deiodinase in peripheral tissues, making the FT3/FT4 ratio a valuable indicator of peripheral sensitivity to THs (7). In this analysis, we observed significant correlations between levels of SFAs and an elevated ratio of FT3/FT4. This indicates that the regulation of serum SFA concentrations may be related to increased peripheral bioavailability of THs, but it is not explicitly determined whether this is a direct regulation or an indirect association. A study involving euthyroid individuals from communities discovered negative associations between FT3/FT4 and serum fatty acid-binding protein 4 (FABP4) (29). FABP4 is a member of the fatty acid-binding protein (FABP) family, which can reversibly bind to long-chain fatty acids, facilitating their transport to specific organelles within cells and participating in the regulation of lipid transport and cellular responses. FABP4 typically exhibits higher affinity and selectivity for long-chain fatty acids compared to albumin (30). Hence, a reduction in FABP4 levels could lead to an increase in circulating FFAs, which may be an intermediary link between FT3/FT4 and SFAs. Previous studies across diverse populations have indicated positive correlations between the FT3/FT4 ratio and the risk of metabolic syndrome, insulin resistance, and dyslipidemia (7, 31). Therefore, it is plausible to consider that the regulation of peripheral TH sensitivity may be one of the mechanisms contributing to metabolic abnormalities. However, further research is needed to gather sufficient direct evidence to support this hypothesis.

The central sensitivity to THs was assessed based on the interaction between FT4 and TSH, measuring pituitary suppression by FT4. TFQI, TT4RI, and TSHI are validated for the assessment (12). In the present study, despite statistically significant correlations between myristic and lignoceric acids with TFQI, and pentadecanoic, margaric acids with TT4RI, the small regression coefficients indicated that the linkage between central sensitivity to THs and the regulatory role of SFAs was not well-defined.

On the other hand, the thyroid gland is recognized as a potential target organ vulnerable to lipotoxic effects. There is a growing body of evidence that links dyslipidemia to impaired thyroid function. An epidemiological study in community populations suggested that individuals with elevated blood lipids had an increased risk of subclinical hypothyroidism (32). Zhao et al. (33) reported that palmitic acid could lead to excessive lipid accumulation in human primary thyrocytes. Exposure to palmitic acid resulted in the downregulation of key proteins involved in TH synthesis, including Tg, sodium iodide symporter, and thyroperoxidase; thereby affecting the synthesis process. Zhang et al. (34) found that high-fat diet-fed rats and palmitate-treated thyroid cells experienced endoplasmic reticulum stress, which compromised Tg and consequently led to impaired thyroid function. Moreover, in the case report, FFAs generated by heparin administration could inhibit the binding of THs to thyroxine-binding globulin (35). This finding seems to contradict the negative correlation we have noted between SFAs and the ratio of FT3/TT3. It should be clearly noted the heparin-induced effect is a short-term alteration due to pharmaceutical intervention, while current data regarding the potential impact of chronic lipotoxicity on the peripheral homeostasis of THs remain limited, thus future research is anticipated to enhance our understanding.

Collectively, there may be bidirectional effects between SFAs and thyroid function. In clinical practice, exploring personalized nutritional patterns based on different states of thyroid function may be beneficial for lipid metabolic health. Moreover, for patients with hypothyroidism or subclinical hypothyroidism, actively improving thyroid function may be helpful in reducing elevated levels of SFAs. Lastly, hypothyroid patients undergoing auxiliary interventions to reduce SFAs may lower the risk of complications associated with both conditions, such as cardiovascular diseases and metabolic syndrome. These hypotheses await validation through well-designed interventional studies.

To our knowledge, this is the first research to reveal the relationship between eleven circulating SFAs and thyroid parameters in American adults. However, some limitations should be acknowledged. First, the cross-sectional design cannot determine a causal link between thyroid parameters and SFA levels. Prospective studies are required to demonstrate causal relationships. Second, socio-economic and lifestyle information were derived from questionnaires, which may introduce reporting bias. Subsequently, since the subsample was selected based on the availability of data on SFAs and thyroid function, the generalizability of the findings may be constrained. Further research is warranted to confirm the relationship among a broader demographic of Americans and in other geographical areas.

## 5 Conclusion

In conclusion, the present study has provided evidence that concentrations of circulating SFAs are correlated with thyroid parameters. Currently, many dietary guidelines advocate restricting SFA consumption for the betterment of overall health. However, it is imperative to acknowledge the impact of hormonal factors, particularly THs, on SFA levels within the body, to develop more precise and targeted intervention strategies. Moreover, there is a need to investigate the fundamental mechanisms and identify possible intervention targets.

## Data Availability

NHANES datasets are publicly available and can be found in online repositories: https://wwwn.cdc.gov/nchs/nhanes.
