# Supplementary material for: The association between circulating saturated fatty acids and thyroid function: results from the NHANES 2011−2012
Source: Front Endocrinol (Lausanne). 2024 Oct 7;15:1405758. doi: 10.3389/fendo.2024.1405758 (PMC11491677; doi:10.3389/fendo.2024.1405758)
Supplement: Supplementary file 1 [file Table1.docx]

Supplementary Material

# TABLE S1 Comprehensive websites detailing laboratory methodologies, quality control and assurance protocols.

| **Analyte** | **Website** |
| --- | --- |
| TSH | https://wwwn.cdc.gov/nchs/data/nhanes/2011-2012/labmethods/thyrod_g_met_tsh.pdf |
| TT4 | https://wwwn.cdc.gov/nchs/data/nhanes/2011-2012/labmethods/thyrod_g_met_tt4.pdf |
| FT4 | https://wwwn.cdc.gov/nchs/data/nhanes/2011-2012/labmethods/thyrod_g_met_free_t4.pdf |
| TT3 | https://wwwn.cdc.gov/nchs/data/nhanes/2011-2012/labmethods/thyrod_g_met_tt3.pdf |
| FT3 | https://wwwn.cdc.gov/nchs/data/nhanes/2011-2012/labmethods/thyrod_g_met_free_t3.pdf |
| TPOAb | https://wwwn.cdc.gov/nchs/data/nhanes/2011-2012/labmethods/thyrod_g_met_thyroid_peroxidase_antibodies.pdf |
| TgAb | https://wwwn.cdc.gov/nchs/data/nhanes/2011-2012/labmethods/thyrod_g_met_thyroglobulin_antibodies.pdf |
| Tg | https://wwwn.cdc.gov/nchs/data/nhanes/2011-2012/labmethods/thyrod_g_met_thyroglobin.pdf |
| FAs | https://wwwn.cdc.gov/nchs/data/nhanes/2011-2012/labmethods/FAS_G_MET.PDF |

TSH: thyroid stimulating hormone; TT4: total thyroxine; FT4: free thyroxine; TT3: total triiodothyronine; FT3: free triiodothyronine; TPOAb: thyroid peroxidase antibodies; TgAb: thyroglobulin antibodies; Tg: thyroglobulin; FAs: fatty acids.

**TABLE S2** Univariate linear regression analyses between variables and SFAs.

| **SFAs** | **Age (Years)** | **Gender** | **BMI category** | **Ethnicity** | **Education** | **PIR category** | **Smoking status** | **Alcohol use** | **Physical Activity** | **Daily calorie intake** | **Diabetes** | **Hypertension** | **ASCVD** | **ALT (U/L)** | **AST (U/L)** | **ALP (U/L)** | **SCr (μmol/L)** | **UIC category** |
| --- | --- | --- | --- | --- | --- | --- | --- | --- | --- | --- | --- | --- | --- | --- | --- | --- | --- | --- |
|  | **β (95% CI), *p* value** | | | | | | | | | | | | | | | | | |
| Capric acid (C10:0) | -0.002 (-0.005,0.001), 0.262 | -0.160 (-0.272, -0.048), 0.005 | 0.033 (-0.106, 0.171), 0.642 | -0.372 (-0.515, -0.229), 0.001 | -0.060 (-0.198, 0.077), 0.388 | 0.130 (-0.001, 0.262), 0.052 | -0.022 (-0.164, 0.121), 0.763 | 0.224 (0.098, 0.351), 0.001 | -0.095 (-0.288, 0.098), 0.335 | 0.143 (0.019, 0.266), 0.023 | 0.027 (-0.114, 0.167), 0.710 | -0.017 (-0.134, 0.099), 0.769 | -0.078 (-0.267, 0.112), 0.423 | 0.006 (0.003, 0.009), 0.001 | 0.004 (0.002, 0.007), 0.002 | 0.003 (0.001, 0.006), 0.011 | -0.001 (-0.002, 0.001), 0.487 | 0.022 (-0.100, 0.144), 0.726 |
| Lauric acid (C12:0) | 0.001 (-0.003, 0.005), 0.559 | -0.141 (-0.282, 0.001), 0.051 | 0.160 (-0.014, 0.333), 0.072 | -0.533 (-0.712, -0.354), 0.001 | -0.159 (-0.332, 0.013), 0.071 | 0.039 (-0.127, 0.205), 0.646 | 0.006 (-0.174, 0.186), 0.949 | 0.030 (-0.130, 0.191), 0.712 | -0.091 (-0.334, 0.152), 0.462 | 0.091 (-0.066, 0.248), 0.255 | 0.172 (-0.004, 0.349), 0.056 | 0.041 (-0.106, 0.187), 0.585 | 0.039 (-0.200, 0.278), 0.748 | 0.005 (0.001, 0.009), 0.018 | 0.002 (-0.002, 0.005), 0.386 | 0.003 (0.000, 0.006), 0.045 | 0.000 (-0.002, 0.002), 0.921 | 0.072 (-0.082, 0.225), 0.358 |
| Myristic acid (C14:0) | 0.003 (0.000, 0.005), 0.057 | -0.116 (-0.213, -0.018), 0.020 | 0.141 (0.022, 0.261), 0.021 | -0.443 (-0.565, -0.321), 0.001 | -0.141 (-0.260, -0.022), 0.020 | 0.019 (-0.096, 0.134), 0.744 | 0.059 (-0.065, 0.183), 0.350 | 0.085 (-0.025, 0.196), 0.131 | -0.071 (-0.239, 0.097), 0.405 | 0.078 (-0.030, 0.186), 0.157 | 0.193 (0.072, 0.314), 0.002 | 0.088 (-0.013, 0.189), 0.088 | 0.047 (-0.118, 0.212), 0.576 | 0.006 (0.003, 0.009), 0.001 | 0.003 (0.000, 0.005), 0.019 | 0.004 (0.001, 0.006), 0.001 | -0.001 (-0.002, 0.001), 0.312 | 0.073 (-0.033, 0.179), 0.177 |
| Pentadecanoic acid (C15:0) | 0.002 (0.000, 0.004), 0.059 | 0.006 (-0.068, 0.080), 0.875 | 0.055 (-0.035, 0.146), 0.232 | -0.378 (-0.468, -0.287), 0.001 | -0.085 (-0.175, 0.005), 0.063 | 0.084 (-0.002, 0.170), 0.055 | -0.015 (-0.109, 0.079), 0.759 | -0.009 (-0.093, 0.074), 0.832 | -0.018 (-0.144, 0.108), 0.779 | 0.037 (-0.045, 0.119), 0.377 | 0.066 (-0.026, 0.158), 0.159 | 0.003 (-0.073, 0.079), 0.935 | -0.035 (-0.159, 0.089), 0.581 | 0.001 (-0.001, 0.003), 0.308 | -0.001 (-0.003, 0.001), 0.220 | 0.002 (0.000, 0.003), 0.065 | -0.001 (-0.002, 0.000), 0.080 | 0.079 (0.000, 0.159), 0.050 |
| Palmitic acid (C16:0) | 0.003 (0.001, 0.004), 0.001 | -0.040 (-0.093, 0.012), 0.134 | 0.046 (-0.019, 0.111), 0.163 | -0.151 (-0.219, -0.084), 0.001 | -0.073 (-0.137, -0.009), 0.026 | -0.013 (-0.076, 0.049), 0.673 | 0.028 (-0.039, 0.095), 0.411 | 0.043 (-0.017, 0.103), 0.157 | 0.029 (-0.062, 0.120), 0.535 | 0.006 (-0.053, 0.065), 0.844 | 0.140 (0.075, 0.205), 0.001 | 0.081 (0.026, 0.135), 0.004 | 0.040 (-0.049, 0.130), 0.377 | 0.003 (0.002, 0.005), 0.001 | 0.002 (0.001, 0.003),0.004 | 0.002 (0.001, 0.003), 0.001 | 0.000 (-0.001, 0.000), 0.344 | 0.018 (-0.039, 0.075), 0.537 |
| Margaric acid (C17:0) | 0.003 (0.002, 0.004), 0.001 | -0.005 (-0.052, 0.043), 0.847 | 0.031 (-0.027, 0.090), 0.293 | -0.127 (-0.188, -0.066), 0.001 | -0.076 (-0.134, -0.018), 0.010 | 0.011 (-0.044, 0.067), 0.686 | -0.008 (-0.069, 0.052), 0.785 | -0.030 (-0.084, 0.024), 0.278 | 0.004 (-0.078, 0.085), 0.930 | -0.005 (-0.058, 0.048), 0.863 | 0.108 (0.049, 0.167), 0.001 | 0.057 (0.007, 0.106), 0.024 | 0.022 (-0.058, 0.103), 0.584 | 0.001 (0.000, 0.002), 0.193 | -0.001 (-0.002, 0.001), 0.403 | 0.001 (0.000, 0.002), 0.010 | 0.000 (-0.001, 0.000), 0.161 | 0.035 (-0.016, 0.087), 0.181 |
| Stearic acid (C18:0) | 0.003 (0.001, 0.004), 0.001 | 0.014 (-0.029, 0.057), 0.518 | 0.042 (-0.010, 0.095), 0.112 | -0.079 (-0.134, -0.024), 0.005 | -0.041 (-0.093, 0.011), 0.122 | -0.010 (-0.060, 0.040), 0.686 | -0.018 (-0.073, 0.036), 0.509 | 0.014 (-0.034, 0.063), 0.569 | 0.000 (-0.073, 0.073), 0.997 | 0.000 (-0.048, 0.047), 0.992 | 0.079 (0.026, 0.132), 0.004 | 0.076 (0.032, 0.120), 0.001 | 0.022 (-0.05, 0.094), 0.548 | 0.003 (0.001, 0.004), 0.001 | 0.002 (0.001, 0.003), 0.001 | 0.002 (0.001, 0.003), 0.001 | -0.001 (-0.001, 0.000), 0.007 | 0.017 (-0.029, 0.063), 0.476 |
| Arachidic acid (C20:0) | 0.002 (0.001, 0.003), 0.001 | 0.091 (0.053, 0.129), 0.001 | 0.041 (-0.007, 0.089), 0.096 | 0.005 (-0.046, 0.055), 0.860 | 0.039 (-0.009, 0.086), 0.113 | 0.012 (-0.033, 0.058), 0.594 | -0.041 (-0.091, 0.009), 0.104 | -0.036 (-0.080, 0.008), 0.109 | 0.036 (-0.030, 0.103), 0.285 | -0.023 (-0.067, 0.020), 0.292 | 0.036 (-0.013, 0.084), 0.152 | 0.048 (0.008, 0.088), 0.020 | -0.039 (-0.105, 0.026), 0.241 | 0.000 (-0.001, 0.001), 0.537 | 0.000 (-0.001, 0.001), 0.506 | 0.001 (0.000, 0.002), 0.014 | -0.001 (-0.001, 0.000), 0.005 | 0.033 (-0.009, 0.075), 0.128 |
| Docosanoic acid (C22:0) | 0.001 (0.000, 0.002), 0.020 | 0.089 (0.050, 0.128), 0.001 | 0.052 (0.003, 0.100), 0.037 | 0.010 (-0.041, 0.060), 0.706 | 0.081 (0.033, 0.129), 0.001 | 0.034 (-0.012, 0.080), 0.149 | -0.065 (-0.115, -0.015), 0.012 | -0.022 (-0.067, 0.023), 0.330 | -0.006 (-0.075, 0.062), 0.855 | -0.003 (-0.047, 0.041), 0.888 | -0.008 (-0.058, 0.041), 0.740 | 0.015 (-0.026, 0.056), 0.463 | -0.094 (-0.161, -0.027), 0.006 | -0.001 (-0.002, 0.001), 0.309 | -0.001 (-0.002, 0.000), 0.144 | 0.001 (0.000, 0.002), 0.079 | -0.001 (-0.001, 0.000), 0.001 | 0.028 (-0.014, 0.071), 0.192 |
| Tricosanoic acid (C23:0) | 0.002 (0.001, 0.003), 0.001 | 0.149 (0.110, 0.188), 0.001 | 0.035 (-0.015, 0.085), 0.172 | 0.019 (-0.034, 0.072), 0.493 | 0.014 (-0.036, 0.064), 0.580 | 0.025 (-0.023, 0.073), 0.312 | -0.094 (-0.145, -0.043),0.001 | -0.053 (-0.099, -0.007), 0.024 | 0.004 (-0.066, 0.075), 0.903 | -0.008 (-0.054, 0.037), 0.714 | -0.011 (-0.062, 0.041), 0.685 | 0.006 (-0.036, 0.048), 0.785 | -0.104 (-0.173, -0.036), 0.003 | -0.001 (-0.002, 0.000), 0.097 | -0.001 (-0.002, 0.000), 0.063 | 0.001 (0.000, 0.002), 0.108 | -0.001 (-0.002, -0.001), 0.001 | 0.035 (-0.009, 0.079), 0.116 |
| Lignoceric acid (C24:0) | 0.001 (0.000, 0.002), 0.020 | 0.014 (-0.025, 0.054), 0.472 | 0.031 (-0.018, 0.079), 0.215 | 0.015 (-0.036, 0.066), 0.559 | 0.051 (0.003, 0.099), 0.037 | 0.017 (-0.029, 0.063), 0.470 | -0.067 (-0.117, -0.017), 0.008 | 0.012 (-0.033, 0.057), 0.604 | -0.027 (-0.095, 0.040), 0.427 | -0.013 (-0.057, 0.031), 0.567 | -0.030 (-0.079, 0.019), 0.234 | 0.003 (-0.038, 0.044), 0.895 | -0.098 (-0.164, -0.032), 0.004 | 0.000 (-0.001, 0.001), 0.564 | 0.000 (-0.001, 0.001), 0.790 | 0.000 (0.000, 0.001), 0.336 | -0.001 (-0.001, 0.000), 0.001 | 0.025 (-0.017, 0.068), 0.247 |
| Sum SFAs | 0.003 (0.001, 0.004), 0.001 | -0.028 (-0.078, 0.022), 0.273 | 0.049 (-0.013, 0.110), 0.119 | -0.143 (-0.207, -0.079), 0.001 | -0.065 (-0.126, -0.004), 0.036 | -0.009 (-0.067, 0.050), 0.772 | 0.015 (-0.049, 0.078), 0.650 | 0.036 (-0.021, 0.092), 0.218 | 0.018 (-0.068, 0.104), 0.679 | 0.008 (-0.048, 0.064),0.781 | 0.125 (0.063, 0.187), 0.001 | 0.076 (0.025, 0.128), 0.004 | 0.031 (-0.054, 0.115), 0.476 | 0.003 (0.002, 0.004), 0.001 | 0.002 (0.001, 0.003), 0.004 | 0.002 (0.001, 0.003), 0.001 | 0.000 (-0.001, 0.000), 0.172 | 0.021 (-0.033, 0.076),0.440 |

SFAs: saturated fatty acids; BMI: body mass index; PIR: poverty index ratio; ASCVD, atherosclerotic cardiovascular disease; ALT: alanine aminotransferase; AST, aspartate aminotransferase; ALP: alkaline phosphatase; SCr, serum creatinine; UIC: urine iodine concentration; CI: confidence interval. SFAs were log-transformed due to non-normally distributed.

**TABLE S3** Multivariate regression analyses of SFAs in relation to TPOAb, TgAb, and Tg.

| **SFAs** | **TPOAb (IU/mL)** | **TgAb (IU/mL)** | **Tg (ng/mL)** |
| --- | --- | --- | --- |
|  | **β (95% CI)** | | |
| Capric acid (C10:0) | 0.055 (0.004, 0.105) ^*^ | 0.065 (-0.024, 0.154) | 0.030 (-0.066, 0.126) |
| Lauric acid (C12:0) | 0.050 (0.003, 0.098) ^*^ | 0.031 (-0.062, 0.123) | 0.035 (-0.066, 0.136) |
| Myristic acid (C14:0) | 0.031 (0.002, 0.060) ^*^ | 0.034 (-0.039, 0.107) | 0.025 (-0.053, 0.103) |
| Pentadecanoic acid (C15:0) | 0.021 (0.000, 0.042) | 0.012 (-0.040, 0.064) | 0.034 (-0.024, 0.092) |
| Palmitic acid (C16:0) | 0.010 (-0.008, 0.028) | 0.008 (-0.026, 0.041) | 0.025 (-0.030, 0.080) |
| Margaric acid (C17:0) | 0.002 (-0.009, 0.012) | -0.015 (-0.043, 0.013) | 0.049 (0.001, 0.098) ^*^ |
| Stearic acid (C18:0) | -0.001 (-0.016, 0.014) | -0.011 (-0.028, 0.006) | 0.043 (-0.006, 0.093) |
| Arachidic acid (C20:0) | 0.014 (0.001, 0.027) ^*^ | 0.003 (-0.022, 0.029) | 0.032 (-0.008, 0.072) |
| Docosanoic acid (C22:0) | 0.007 (-0.005, 0.018) | 0.011 (-0.008, 0.030) | 0.011 (-0.022, 0.045) |
| Tricosanoic acid (C23:0) | 0.017 (0.008, 0.025) ^*^ | 0.020 (0.005, 0.036) ^*^ | 0.016 (-0.015, 0.027) |
| Lignoceric acid (C24:0) | 0.009 (0.001, 0.018) ^*^ | 0.016 (-0.001, 0.034) | 0.006 (-0.026, 0.038) |
| Sum SFAs | 0.009 (-0.008, 0.026) | 0.005 (-0.026, 0.036) | 0.029 (-0.025, 0.082) |

SFAs: saturated fatty acids; TPOAb: thyroid peroxidase antibodies; TgAb: thyroglobulin antibodies; Tg: thyroglobulin; * *p* < 0.05. Age, gender, BMI category, ethnicity, education, PIR, smoking status, alcohol use, daily calorie intake, diabetes, hypertension, ASCVD, ALT, AST, ALP, SCr, and UIC were adjusted. SFAs and thyroid function measures were log-transformed due to non-normally distributed.
